# Asynchronous Control of P300-Based Brain–Computer Interfaces Using Sample Entropy

**DOI:** 10.3390/e21030230

**Published:** 2019-02-27

**Authors:** Víctor Martínez-Cagigal, Eduardo Santamaría-Vázquez, Roberto Hornero

**Affiliations:** Biomedical Engineering Group, E.T.S.I. Telecomunicación, University of Valladolid, Paseo de Belén 15, 47011 Valladolid, Spain; eduardo.santamaria@gib.tel.uva.es (E.S.-V.); robhor@tel.uva.es (R.H.)

**Keywords:** sample entropy, multiscale entropy, brain–computer interfaces, asynchrony, event-related potentials, P300-evoked potentials, oddball paradigm

## Abstract

Brain–computer interfaces (BCI) have traditionally worked using synchronous paradigms. In recent years, much effort has been put into reaching asynchronous management, providing users with the ability to decide when a command should be selected. However, to the best of our knowledge, entropy metrics have not yet been explored. The present study has a twofold purpose: (i) to characterize both control and non-control states by examining the regularity of electroencephalography (EEG) signals; and (ii) to assess the efficacy of a scaled version of the sample entropy algorithm to provide asynchronous control for BCI systems. Ten healthy subjects participated in the study, who were asked to spell words through a visual oddball-based paradigm, attending (i.e., control) and ignoring (i.e., non-control) the stimuli. An optimization stage was performed for determining a common combination of hyperparameters for all subjects. Afterwards, these values were used to discern between both states using a linear classifier. Results show that control signals are more complex and irregular than non-control ones, reaching an average accuracy of 94.40% in classification. In conclusion, the present study demonstrates that the proposed framework is useful in monitoring the attention of a user, and granting the asynchrony of the BCI system.

## 1. Introduction

Brain–computer interfaces (BCI) are able to detect users’ intentions from brain signals and convert them into artificial commands that control an external device. BCI applications are intended to replace, restore, enhance, supplement, or improve the natural central-nervous-system activity of the user [1]. Such purposes make BCI systems especially suited for improving the quality of life of motor-disabled people, reducing their dependence, and favoring their social and labor integration. These disabilities may be caused by traumas, neurodegenerative diseases, muscle disorders, or any illness that impairs the neural pathways that control muscles or the muscles themselves [2]. Although there are several ways to monitor the brain activity of a user, electroencephalography (EEG) is generally used due to its noninvasiveness, portability, and low cost. Therefore, electric brain activity is recorded by placing a set of electrodes on the user’s scalp [2].

Since a user’s intentions are not directly reflected in the raw EEG signal, BCI systems rely on the processing of measurable changes related to cognitive tasks, known as control signals [3]. Event-related potentials, such as P300 responses, are commonly used to assure the robustness of the system regardless of disability. P300-evoked potentials are the brain’s natural responses to infrequent and significant stimuli, elicited approximately 300 ms after their onset [2,3]. Owing to their exogenous nature, previous training is not necessary, which makes a P300-based BCI suitable for any person who presents a certain degree of gaze control. In this sense, the row-col paradigm (RCP), a particularization of the oddball visual paradigm, is the most common setup to aid users in spelling words or commands [4]. In this paradigm, a matrix containing alphanumeric characters or commands is displayed. Users just need to focus their attention on the desired command while the matrix’s rows and columns randomly flash. Whenever the target’s row or column is intensified, P300 potential is generated. Hence, the desired command can be determined by identifying when these potentials have been elicited [2,4].

The RCP is a synchronous process. Due to continuous stimulation, the system makes a selection even if the user does not pay attention to the visual stimuli [5]. In a real application, it is desirable that users voluntarily decide when they want to select a command and when they do not. For instance, if the purpose of the BCI system is to provide disabled users with an assistive tool to surf the Internet, the application should be able to detect if the user wants to select a navigation command or, by contrast, to calmly read a webpage or watch a video [6]. A conventional synchronous BCI could not monitor users’ attention; thus, it continues selecting random commands while users ignore the visual stimulation. Therefore, the default synchronous mode of the RCP severely restricts the applicability of a BCI system in a real environment, requiring an external supervisor or the inclusion of a read-mode command that pauses the RCP for a fixed time. In order to overcome this limitation, the system should be able to discern between the control state (i.e., when users pay attention to the stimuli) and the non-control state (i.e., idle state, when users ignore the stimuli). In other words, the RCP-based system must become an asynchronous application. In recent years, several efforts have been made to achieve real asynchronous control [7]. Most related P300-based BCI studies rely on a threshold derived from classifiers’ scores, which are expected to be higher in the control state than in the non-control state. These scores were obtained from support vector machines (SVM) [8,9,10] or linear discriminant analysis (LDA) [6,11,12,13,14,15,16] classifiers using downsampled raw signals from the stimuli onset as features [3,5,6,8,9,16,17,18,19,20]. Aydin et al. also used classifier labels instead of scores to design different criteria to identify the idle state [20]. Other studies proposed spectral features to detect both states, such as relative powers [9,17,21] or sums of spectral components [5]. Among these complementary metrics, recent studies have proposed modified BCI frameworks. Panicker et al. and Li et al. proposed novel asynchronous paradigms that involve steady-state visually evoked potentials (SSVEP) and P300 responses at the same time, using SSVEP to identify the idle state, and P300 responses to determine the desired commands in real time [9,17]. Breitwieser et al. provided asynchronous control in a tactile-based BCI system to detect both steady-state somatosensory evoked potentials (SSSEP) and transient event-related potentials (tERP) [13]. Lastly, Yu et al. presented a hybrid system that manages asynchronous control using motor imagery, while an RCP matrix controls the command selection [18,22].

Despite the recent interest in providing asynchronous control in RCP-based BCI systems, to the best of our knowledge, entropy metrics have not yet been explored. In this context, we hypothesize that different entropy metrics could provide insight into the dynamics of attended and nonattended EEG signals, providing complementary information to discern between both states. Particularly, multiscale entropy (MSE) based on sample entropy (SampEn) has demonstrated to be effective in estimating the complexity and regularity of physiological time series [23,24,25,26]. Thus, differences between the regularity of control and non-control EEG signals could be expected to be found. Therefore, the present study has a twofold purpose: (i) to characterize control and non-control states by examining the regularity of EEG signals; and (ii) to assess the efficacy of a scaled version of SampEn to provide asynchronous control in P300-based BCI systems.

## 2. Materials and Methods

EEG signals show high intersubject variability and, thus, BCI systems must be optimized for each subject [2,3,4,6,19]. The amplitude and latency of P300 responses have been demonstrated to vary depending on individual differences, such as age or personality, pharmacological aspects, or even clinical disorders [27]. Therefore, channel and feature selection methods, as well as classifiers, are always optimized in the first session of each user. According to this rationale, classifiers of the present study are separately trained and tested, returning a final accuracy for each subject.

**Figure 1:**
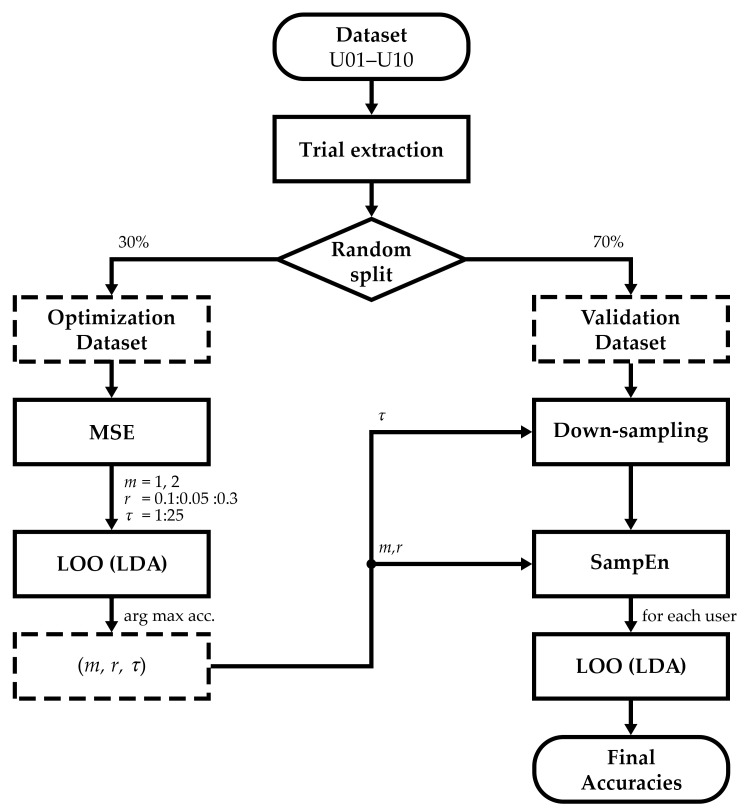
Methodological flowchart of the study. Once trials were extracted, the dataset was divided into optimization and validation sets. The former was intended to optimize a global combination of hyperparameters *m*, *r*, and τ; in the latter, these values were applied to compute the final accuracy of each user.

The methodological structure of the study is depicted in the flowchart of Figure 1. Once the dataset was registered and preprocessed, it was randomly divided into optimization (30%) and validation (70%) datasets. The optimization set was used to characterize the asynchronous states and find an optimal combination of the required hyperparameters that could work with all subjects. These global values were thereafter used to test the validation set for each user and assess the ability of the framework to discriminate between control and non-control states. Training and testing were employed under a leave-one-out (LOO) procedure, intended to provide a final accuracy for each user.

### 2.1. Dataset and Experimental Protocol

**Figure 2:**
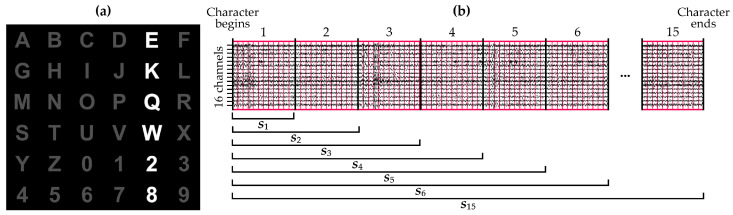
(**a**) Row-col paradigm matrix employed in this study. Currently, the fifth column is being flashed; (**b**) Trial extraction procedure of a single character in function of the number of sequences. Considering the *i*-th sequence, trial $s_{i}$ is composed of the signal from the first sample to the last onset of the *i*-th sequence. Therefore, a total of 15 trials were extracted for each character.

Ten control subjects (mean age 25.7±3.09 years; 6 males, 4 females) were included in this study. All of them gave their informed written consent to participate. Subjects were asked to perform spelling tasks using a 6×6 RCP matrix in two different sessions, shown in the Figure 2a. In the RCP paradigm, the matrix’s rows and columns randomly flash [4]. Users, who were asked to stare at the desired command, elicited P300 responses when the row and the column that contained that command were illuminated. Therefore, the desired command could be determined by identifying these responses [2,4]. In order to favor their concentration, users were also asked to count how many times the desired command flashed. For each user, a total of 120 characters were spelled. Half of them were recorded following the aforementioned protocol, intended to get the signal in the control state. For the other half, users were asked to read a text while ignoring the flashings. Hence, these characters were intended to record the non-control state. Note that a character comprised 15 sequences (i.e., repetitions) of flashings, where a sequence comprises all flashes that are required to highlight each row and column of the matrix. Each flashing lasted 75 ms, followed by an interstimuli interval of 100 ms. EEG signals were recorded using a g.USBamp amplifier (g.Tec, Austria) with a sampling rate of 256 Hz. In all, 16 active electrodes were placed on Fz, F3, F4, Cz, C3, C4, CPz, Pz, P3, P4, POz, PO3, PO4, PO7, PO8, and Oz, using Fpz as a ground and the earlobe as a reference according to the International 10–20 System distribution [28]. Since P300 responses are thought to be more prominent over the visual cortex and related with cognitive processing, electrodes were mainly placed on the occipital and parietal lobes [2,3].

As a preprocessing stage, a band-pass filter in the range of 0.1–30 Hz and a common average reference (CAR) spatial filter were applied to the raw signals [2,6,19]. Afterward, trials were extracted from the EEG signals for each channel following the procedure that is depicted in Figure 2b. As can be seen, each trial integrates the signal from the first sample to the last onset that belongs to the maximum considered sequence. For instance, the *i*-th trial comprises the raw signal of all electrodes since the very first recording sample of the character until the end of the *i*-th sequence. Then, the dataset was randomly split up into optimization (30%) and validation (70%) sets. These ratios were maintained for each user, resulting in a total of 36 characters for the optimization set and 84 characters for the validation set per user. It is noteworthy that both sets were also balanced, including the same number of control and non-control characters of each user.

### 2.2. Optimization Stage

The optimization stage was intended to find a global combination of hyperparameters that favor the discrimination between control and non-control states for all users. To this end, features were first extracted by means of MSE, and then classified with an LDA following a LOO procedure. As indicated in Figure 1, the combination of parameters was finally selected under a criterion of maximum performance.

MSE is a well-known nonlinear method that estimates the complexity of a signal according to entropy changes along multiple time scales [24]. The algorithm sequentially computes the entropy of a coarse-grained version of the original signal, providing information about its dynamical structure [24,25]. If MSE is applied on two different time series, and one of them provides higher entropy values for most scales, it is considered to be more complex [24,25]. Typically, the τ-th scaled coarse-grained signal is obtained by averaging the samples of the time series inside consecutive but nonoverlapped segments of length N/τ, where *N* denotes the length of the signal [24]. However, it was shown that this procedure may cause aliasing and, thus, spurious components in the low-frequency range [26,29]. In order to overcome this limitation, we decimated the original signal by a factor of τ. That is, high frequencies were reduced with a low-pass least-squares linear-phase FIR filter, followed by a downsampling procedure that only kept every τ-th sample [26,29]. Therefore, the MSE algorithm computes the entropy of each signal as a function of τ from the original time series (i.e., τ=1), to the highest considered scale (i.e., τ=25) [26].

SampEn is a single-scale entropy measure that estimates the irregularity of one-dimensional temporal signals, assigning higher values to series that show larger degrees of disorder [23]. Compared to the approximate entropy algorithm, SampEn eliminates the inherent bias caused by self-matching and provides a result less dependent on signal length [23]. For this reason, SampEn has been widely used to compute the MSE and its variants [26]. Briefly, the algorithm provides a conditional probability measure that quantifies the likelihood that a template of *m* consecutive samples, which already matches another sequence, still matches it if their lengths are increased in one sample [26]. Therefore, SampEn is defined as:

(1) $$SampEn\left(m,r,N\right)=\lim_{N\to \infty }-ln\frac{A^{m}\left(r,N\right)}{B^{m}\left(r,N\right)},$$

where *m* is the embedding dimension, *r* is the tolerance factor, *N* is the length of the signal, and $A^{m}\left(r,N\right)$ and $B^{m}\left(r,N\right)$ are the probabilities of template matching for m+1 and *m* points, respectively. Considering a time series $x=[x_{1},x_{2},\cdots ,x_{N}]$, where template vectors of length *m* are defined as $x_{m}\left(i\right)=\left[x_{i},x_{i+1},\cdots x_{i+m-1}\right]$, a match between two templates $x_{m}\left(i\right)$ and $x_{m}\left(j\right)$ occur if the distance between them is less than a certain tolerance value: $d[x_{m}\left(i\right),x_{m}\left(j\right)]<R$. Although there are a variety of distance measures, Chebyshev distance is commonly used [23]. Moreover, tolerance is used to be dependent of the standard deviation of the signal (i.e., $R=r\cdot \sigma_{x}$) [23,26]. In practice, SampEn is estimated as follows:

(2) $$SampEn\left(m,r,N\right)=-ln\left(\frac{N-m+1}{N-m-1}\cdot \frac{A}{B}\right),$$

where *A* and *B* are the total number of templates of lengths m+1 and *m* that meet the distance criterion for each different combination of *i* and *j*, given i≠j, respectively. Since the total number of possible templates of lengths m+1 and *m* along the signal are N−m+1 and N−m−1, respectively; normalization is also applied to correct the estimation. As a result of the approximation of Equation (1), the variance of the entropy estimator grows as the length of the signal decreases [26]. Therefore, the longer the signal length, the more reliable the outcome is. As a general rule of thumb, the estimation of SampEn is considered accurate if $N\geq 10^{m}$ [23,26].

**Figure 3:**
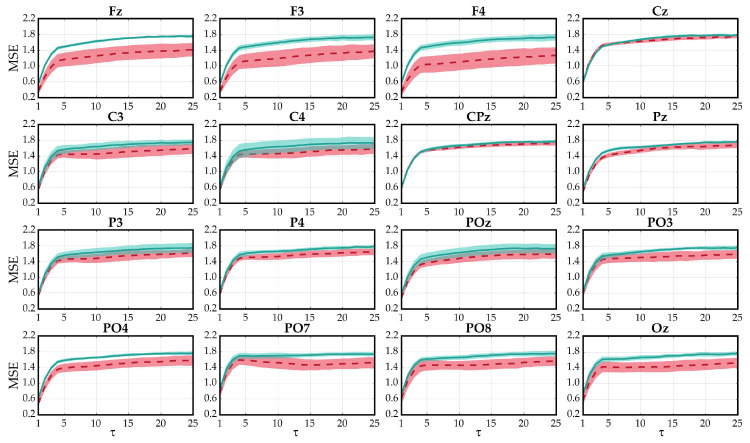
Multiscale sample entropy values from the optimization dataset corresponding to U05 across channels. Solid lines indicate the average values for control (blue) and non-control (red) trials, whereas shaded areas indicate standard deviation. Embedding dimension and tolerance parameters were fixed to m=1 and r=0.3, respectively.

MSE using a SampEn estimator was then applied to the optimization dataset. Hyperparameters were varied according to common ranges widely used in physiological signals: embedding dimensions m=1,2; tolerances *r* from 0.1 to 0.3 in steps of 0.05; and scales τ from 1 to 25 [23]. Scales that did not meet the Richman & Moorman criterion (i.e., $N\geq 10^{m}$) were not computed [23]. Since entropies should be estimated in one-dimensional signals, MSE was calculated for each channel, returning a final value per channel and trial. Note that trials were extracted following the procedure described in Section 2.1, computing the MSE using different number of RCP sequences, from 1 to 15. Figure 3 depicts the MSE results of the user U05 for illustrative purposes.

In order to determine a common optimal combination of τ, *m*, and *r* for all users, an LOO procedure was performed. LOO cross-validation is a deterministic technique that estimates how the results of a statistical model generalize to an independent dataset [30]. The algorithm sequentially classifies an observation with a model trained with the remaining ones. This process is repeated until all observations have been tested, returning the average of the prediction outcomes as an estimation of the accuracy [30]. In this case, the LOO procedure integrated an LDA that classified control versus non-control observations, where MSE results of each channel were included as features. The accuracies for all trials, $s_{1},s_{2},\cdots ,s_{15}$, were averaged in order to get a single accuracy value for each combination of τ, *m*, and *r*. Lastly, the combination of hyperparameters that reached maximum accuracy was thereafter considered optimal. Owing to the mix of users that composes the optimization dataset, the optimal *m*, *r*, and τ are expected to work properly regardless of subject.

### 2.3. Validation Stage

The validation stage was intended to assess the performance of the proposed framework to determine the state of the user and achieve asynchronous control of the system. As can be noticed, since MSE was not computed to consider any geometric feature of the curve, but to determine an optimal scale τ, there is no point in calculating the MSE in the validation dataset. Instead, validation signals for each user are first downsampled to optimal scale τ. Afterward, features are the SampEn outcomes of each channel using optimal *m* and *r* hyperparameters. An LDA-based LOO procedure is finally used to estimate the accuracy of the classification per user and sequence.

## 3. Results

**Figure 4:**
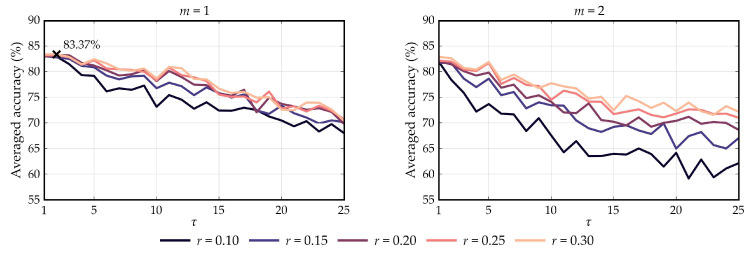
Accuracy results of the optimization stage in function of different values of embedding dimension *m*, tolerance *r*, and scale τ. Optimal combination of hyperparameters is marked with a cross, which corresponds to m=1, r=0.3, and τ=2.

**Figure 5:**
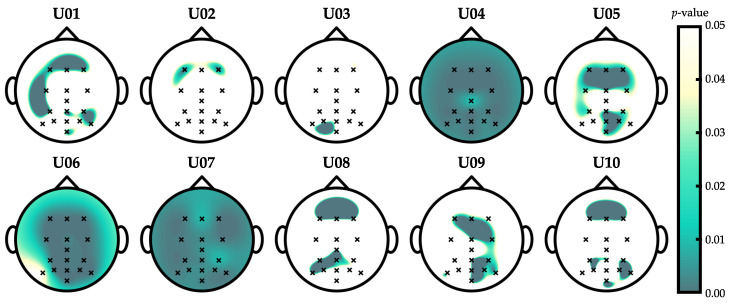
Wilcoxon signed-rank test *p*-values that show significant differences (i.e., from 0 to 0.05) between control and non-control SampEn features in the optimization dataset. Hyperparameters were fixed to their optimal values. Note that *p*-values were adjusted using the Benjamini–Hochberg False Discovery Rate (FDR) step-up procedure.

Optimization results are depicted in Figure 4. As can be seen, the estimated accuracies show a decreasing tendency as the scale increases regardless of embedding dimension. According to the maximum-accuracy criterion, the optimal combination of hyperparameters was found to be m=1, r=0.3, and τ=2. Figure 5 depicts the spatial distribution of the significant differences that were found between control and non-control SampEn features in the optimization dataset (Wilcoxon signed rank test), using the aforementioned optimal parameters. It is noteworthy that the Benjamini–Hochberg False Discovery Rate (FDR) correction was applied to counteract the problem of multiple comparisons (i.e., 16 channels) [31]. As shown, significant differences were mainly found in prefrontal and occipital electrodes.

**Figure 6:**
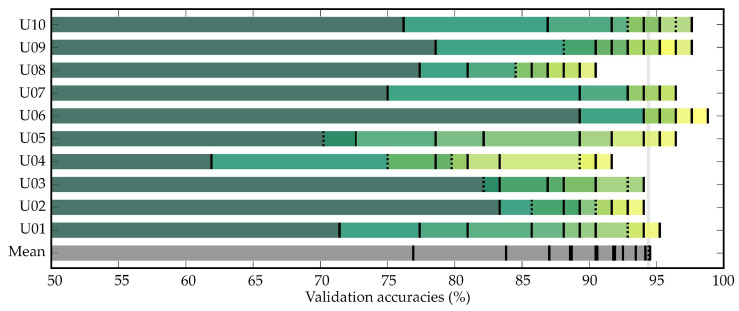
Cumulative testing accuracies (control vs. non-control) as sequences increase for each subject. Lines indicate the number of sequences, where a solid line implies an increase and a dashed line implies a decrease of accuracy.

The results of the validation stage are displayed in Table 1 and Figure 6. The proposed framework reached a mean accuracy of 94.40%±2.81% across subjects for 15 sequences. Figure 6 depicts the cumulative testing accuracies (control vs. non-control) as the number of sequence increases for each subject. As can be seen, users generally showed an improvement in performance as more sequences are considered, reaching more than 90% of accuracy in every case. In order to guarantee the application of the proposed framework in real time, computational cost analysis is shown in the Table 2, which details the required time to compute the SampEn algorithm using different numbers of sequences. Analysis was made using an Intel Core i7-7700 CPU @ 3.60GHz (32 GB RAM, Windows 10, MATLAB^®^2018a), performing an average of 1000 iterations of the algorithm.

## 4. Discussion

Significant differences were found between control and non-control states using features derived from MSE and SampEn. Since the depicted behavior of Figure 3 is representative of all subjects, SampEn values of control states were slightly higher than those obtained in non-control states. Moreover, this behavior is almost constant as scales increase (i.e., amount of decimation). The MSE values of both states show an increasing trend until τ=4, steadying themselves after that point. On the one hand, this tendency implies that attending to an RCP paradigm produces more irregular signals than ignoring the stimuli [23]. On the other hand, although both states show a similar response to dynamical changes in different scales, control signals present a steeper slope. Therefore, control-state signals can be considered more complex than non-control ones because they are more irregular in most scales [24,25]. It is also noteworthy that SampEn values of nonattending signals become more unstable as the scale increases, raising the standard deviation. By contrast, attending signals are generally more defined, showing smaller values of standard deviation.

Regarding the optimization stage, it is noteworthy that the performance of the method depends on the hyperparameters. Although MSE values do not seem to be affected by tolerance, the embedding dimension and the scale play an important role in the proposed framework. As can be seen in Figure 4, performance showed a decreasing tendency as τ increased, regardless of the value of *r*. As aforementioned, the standard deviation of non-control MSE values increases with τ, while control MSE values remain almost constant. Hence, the decrease in performance for high scales is expected. Although accuracy values when m=1 are not appreciably affected by *r*, performance decays as *r* decreases when m=2. This behavior is also expected according to the SampEn algorithm, since higher tolerance values increment the probability of finding template matchings and, thus, increasing variability between different runs of the LOO procedure. In summary, the optimal embedding dimension and tolerance parameters were found to be m=1 and r=0.3, respectively, in accordance with previous studies that used physiological signals [23]. Concerning the optimal τ=2 scale, it is equivalent to reducing the sampling rate of the EEG signal by half before applying the SampEn algorithm. This procedure can be addressed as a feature-extraction stage that is common in P300-based BCI studies [3,5,6,8,9,16,17,18,19,20].

In this context, the estimation of SampEn could be considered accurate when signal length is greater than ten to the power of the embedding dimension (i.e., $N\geq 10^{m}$) [23,26]. According to Figure 2, signal length depends on the number of sequences that are considered, as well as on the amount of decimation. Since this limitation takes into account the amount of raw samples, the maximum number of scales that can be computed in a reliable way are thus limited by the number of sequences, the sampling rate, the stimuli duration, the number of commands and, in general, by any parameter that affects the duration of a character trial. In a P300-based BCI common setup, this constraint is not usually present for a high number of sequences (i.e., $N_{s}$), but it is recommended to compute the maximal scale in each situation. In our study, the entire number of 25 scales could be computed if $N_{s}>4$, reaching a maximum of four scales using only one sequence. Owing to fixing the optimal scale to τ=2, the constraint did not even limit the number of sequences in our case.

Topographic results show significant differences for almost all users between the entropy values of control and non-control states, mainly in the prefrontal lobe. The prefrontal cortex is commonly associated with planning complex cognitive behavior, personality expression, decision making, and selective attention [32]. The latter is consistent with the oddball task, which implies a constant attention of the user to identify the target stimuli among other background stimuli [3]. In fact, it was demonstrated that visual oddball tasks produce hemodynamic changes in the dorsolateral prefrontal cortex, associated with the mapping of stimuli to responses (e.g., response strategies) [33]. Moreover, a recent study suggested that complex processes such as memory, attention, or decision making are linked to the elicitation of the P300 component, which could be modulated by frequency dynamics [34]. There are also slight differences in the occipital lobe, which comprises most of the anatomical region of the visual cortex. Neurons of the primary visual cortex fire action potentials when visual stimuli appear in the receptive field [35]. It is therefore expected that a higher number of neurons are activated in the control state, when a user not only perceives the target stimuli, but also repetitive flickering stimuli. The task elicits P300-evoked potentials in the parietal cortex when target stimuli are processed [2,3]. However, since we extract features using the entire raw control EEG signal, P300 are surpassed by nontarget stimuli. Recent studies suggest that peripheral flickering stimuli in the RCP task produce SSVEP responses [5,17,21], which propagate from occipital to prefrontal electrodes [36]. Note that these topographic results measure significant differences between the irregularity of control- and non-control-state EEG signals. According to previous analysis, attending to a RCP task should activate a greater number of neurons than ignoring the stimuli, spreading electrical activity across the frequency spectrum. Therefore, entropy measures follow that spectral activation, increasing the irregularity of the control signals.

One of the most crucial obstacles of BCI systems is to find methods that can be applied in real time. In relation to this, we consider important to analyze the potential of the proposed framework to determine the asynchronous state upon which a character is selected. As indicated in Table 2, the maximal computational time of the SampEn algorithm is approximately 197 ms using 15 sequences. Since most P300-based BCI studies use pauses of at least two seconds after each character, the computational cost of the proposed framework is perfectly acceptable [6,16,19,37].

Concerning the validation stage, Figure 6 and Table 1 show an increasing tendency of the final accuracies for all subjects as the number of sequences increases. Therefore, it is clear that the proposed asynchrony approach is dependent on the length of the signals, reaching an average accuracy of 94.40% for all subjects using 15 sequences. In particular, all subjects except U04 and U08 reached more than 90% accuracy using nine sequences. Furthermore, U06, U07, and U09 were even able to reach it using only three sequences. Even though the increasing tendency is clear for all subjects, the slope appreciably varies among them. Some users present a sequential increase (e.g., U01, U05, U08, U09), while others reach a standstill (e.g., U03, U10). These results reinforce the fact that it is important to perform individual calibrations and separately optimize BCI applications to each subject [2,3,19].

Table 3 depicts a comparison between previous asynchronous P300-based state-of-the-art applications. As shown, most of them follow a thresholding approach to discern between control and non-control states [5,6,8,9,11,12,13,14,15,16,20,21]. These thresholds are usually derived from receiver operating characteristic (ROC) curves that were fed using output scores of SVM [8,9] or LDA [5,6,11,13,15,16] classifiers. Note that these classifiers use downsampled raw signals from the stimuli onset as input features [3,5,6,8,9,16,17,18,19,20]. Since they were trained in a calibration session to detect P300 responses, these studies hypothesize that output scores of non-control characters are lower than those spelled in the control state. Therefore, the classifier that is intended to detect the P300 responses is also intended to discern between both asynchronous states. Notwithstanding their usefulness as computationally simple solutions, these approaches entail a clear drawback. Owing to the high intersession variability of the EEG signals, classifier weights should be updated from time to time to assure suitable performance [2,3,6,10,16]. Since threshold values depend on classifier scores, they are no longer useful if these weights are modified. Hence, additional control and non-control characters should be recorded in order to update the thresholds, which would entail a great amount of time. Other approaches add complementary spectral features [5,21] or implement hybrid paradigms [9,17,18] to develop filter methods that are independent of the P300 classifier. Some of the hybrid paradigms superimpose the RCP oddball technique, intended to generate P300 responses, with a flickering visual effect, intended to generate SSVEPs when users are paying attention to the visual stimuli [9,17]. Therefore, asynchrony is handled by the detection of SSVEPs using relative powers: control state if SSVEPs are present, non-control state if SSVEPs are missing [9,17]. Pinegger et al., and Ma & Qiu also used SSVEP detection techniques to reach asynchronous control, but their approach is utterly different [5,21]. By contrast, they hypothesized that inherent RCP flashings also generate residual SSVEP components when the stimuli are displayed using a constant rate. These components were identified in the frequency spectrum, providing supplementary features to the LDA scores [5]. Finally, it is also worthy to mention the contribution of Yu et al., who implemented a hybrid approach to reach a semiasynchronous BCI application [18]. Users activated the RCP flashings by regulating their cortical activity through motor imagery. However, stopping RCP was handled by a “stop” command, which increases the required time to manage the asynchrony and makes the system more demanding. Since the vast majority of these previous studies were intended to provide an asynchronous assistive application, instead of just evaluating a novel method to reach asynchronous control, the provided accuracies reflect the final performance of the system. In other words, results depict the performance of the system to predict correct characters, while ignoring those than are considered non-control. Unfortunately, control versus non-control accuracies are not reported and, thus, quantitative and statistical comparisons cannot be performed with the present study. Despite this issue, it is noteworthy that, to the best of our knowledge, there are no studies that have previously investigated the ability of entropy-based features to discern between both asynchronous states. Moreover, since our approach is independent of classifier, weights updates do not affect asynchronous management, avoiding the need to record extra EEG signals [5,6,8,9,11,12,13,14,15,16,20]. We also believe that further endeavors could be aimed at complementing our proposed entropy features with SSVEP-based ones, which could presumably improve the final performance of asynchronous P300-based BCI systems [5,21].

Owing to these outcomes, several insightful implications can be derived. First, it was demonstrated that a scaled version of SampEn can follow the dynamic changes of control and non-control EEG signals, providing a useful tool to monitor the attention of the user. Furthermore, the proposed framework is not only able to work in real time for P300-based BCI systems, but also may be considered as a filter method. In other words, the metric is independent of the classifier that determines the selected command, in contrast with previous approaches [5,6,8,9,11,12,13,14,15,16,20,21]. Since our proposal does not rely on the classifier’s scores, the command-oriented classifier can be updated without requiring a further training of the asynchrony method. Moreover, both states were also analyzed in this study, showing that control-state signals are more irregular and complex than non-control ones. Finally, a combination of user-independent hyperparameters were determined. To summarize, it was demonstrated that the proposed SampEn-based framework is suitable for providing asynchronous control in P300-based BCI systems.

In spite of these results, the present study has several limitations. The proposed framework only employed temporal features derived from the SampEn algorithm to classify between control and non-control states. The performance of this approach could be extended in the future by integrating complementary spectral features in order to improve its performance [5,9,13,17]. It is also noteworthy that the global combination of hyperparameters was defined using 10 control subjects who are not the target users of BCI systems. A future endeavor should be aimed at incrementing the database with both control and motor-disabled users in order to improve the generalization of these results. Furthermore, the variability of the optimal hyperparameters was not addressed in this study. Finally, it should be noted that the validation stage was applied under an LOO procedure. Although this method is excellent to estimate the performance of a statistical model, it requires more training trials in each iteration than those that are commonly used in practice. Moreover, owing to the limited number of subjects and characters in the database, optimization could not be performed using different users than in the validation procedure.

## 5. Conclusions

In this study, differences between control and non-control signal was analyzed using entropy metrics. Furthermore, a method to discern between both states and provide an asynchronous control of a P300-based BCI has been proposed. Dataset was composed of the EEG signals of ten healthy subjects who were asked to perform spelling tasks in a row-col paradigm, attending and ignoring the stimuli. Signals were then subdivided into optimization and validation sets. The former was used to determine a common optimal combination of hyperparameters by applying MSE features in a LOO procedure. These parameters were thereafter fixed at m=1, r=0.3, and τ=2 for all subjects. Then, the latter was used to test the ability of a scaled version of SampEn to characterize both states. Multiscale analysis results showed that control signals are more irregular and complex than non-control ones, regardless of scale. These features were also demonstrated to be suitable for classifying both states, reaching an average accuracy of 94.40%. From the experimental outcomes of this exploratory research, we conclude that: (i) MSE measures could follow the dynamic changes of control and non-control signals; (ii) the optimal combination of hyperparameters favors the discrimination between both states for all control subjects; (iii) the proposed framework has the potential to provide asynchronous control with high accuracies; and (iv) the computational cost of the method is negligible, reaching real-time processing.

## Figures and Tables

**Table 1:** Testing accuracies of control vs. non-control states for each subject in function of the number of sequences.

$N_{s}$	1	2	3	4	5	6	7	8	9	10	11	12	13	14	15
**U01**	71.43%	77.38%	80.95%	85.71%	88.10%	88.10%	89.29%	90.48%	94.05%	94.05%	92.86%	92.86%	94.05%	95.24%	94.05%
**U02**	83.33%	88.10%	89.29%	85.71%	89.29%	89.29%	91.67%	91.67%	91.67%	90.48%	92.86%	91.67%	92.86%	94.05%	92.86%
**U03**	83.33%	82.14%	88.10%	83.33%	86.90%	90.48%	88.10%	90.48%	94.05%	92.86%	92.86%	92.86%	92.86%	92.86%	92.86%
**U04**	61.90%	78.57%	80.95%	75.00%	75.00%	75.00%	80.95%	80.95%	79.76%	80.95%	83.33%	90.48%	89.29%	91.67%	91.67%
**U05**	72.62%	70.24%	72.62%	78.57%	78.57%	82.14%	89.29%	89.29%	91.67%	91.67%	91.67%	94.05%	95.24%	96.43%	96.43%
**U06**	89.29%	94.05%	96.43%	96.43%	95.24%	94.05%	96.43%	95.24%	94.05%	95.24%	96.43%	96.43%	96.43%	97.62%	98.81%
**U07**	75.00%	89.29%	92.86%	95.24%	96.43%	96.43%	95.24%	95.24%	92.86%	94.05%	95.24%	95.24%	96.43%	96.43%	95.24%
**U08**	77.38%	80.95%	85.71%	86.90%	86.90%	88.10%	86.90%	84.52%	89.29%	89.29%	86.90%	89.29%	90.48%	89.29%	89.29%
**U09**	78.57%	90.48%	91.67%	88.10%	94.05%	90.48%	92.86%	95.24%	95.24%	92.86%	95.24%	95.24%	97.62%	95.24%	96.43%
**U10**	76.19%	86.90%	91.67%	95.24%	95.24%	92.86%	94.05%	92.86%	95.24%	97.62%	97.62%	96.43%	96.43%	96.43%	96.43%
**Mean**	**76.90%**	**83.81%**	**87.02%**	**87.02%**	**88.57%**	**88.69%**	**90.48%**	**90.60%**	**91.79%**	**91.90%**	**92.50%**	**93.45%**	**94.17%**	**94.52%**	**94.40%**
**SD**	7.58%	7.23%	7.11%	7.13%	7.23%	6.18%	4.59%	4.74%	4.61%	4.52%	4.38%	2.46%	2.77%	2.58%	2.81%

$N_{s}$ indicates number of sequences.

**Table 2:** Computational cost in milliseconds of the sample entropy algorithm in function of the number of sequences.

$N_{s}$	1	2	3	4	5	6	7	8	9	10	11	12	13	14	15
**Mean**	0.82	3.46	8.24	14.64	22.57	32.51	43.66	54.92	69.70	86.33	104.87	125.24	146.41	170.58	196.78
**SD**	0.99	0.28	0.82	1.03	1.40	2.00	3.10	3.30	3.84	4.69	5.56	5.96	6.50	7.20	8.64

$N_{s}$ indicates the number of sequences. These results are obtained after running the sample entropy algorithm 1000 times.

**Table 3:** Comparison between previous asynchronous P300-based brain–computer interface (BCI) applications.

Study	Control Signal	Experimental Paradigm	Asynchrony Technique	No. Subjects
Zhang et al., 2008 [8]	P300	Single cell	ROC thresholding using SVM scores	4 CS
Panicker et al., 2010 [17]	P300 and SSVEP	Hybrid: RCP-based	Detection of SSVEPs using relative peak amplitude in PSD	10 CS
Aloise et al., 2011 [11]	P300	RCP	ROC thresholding using LDA scores	11 CS
Li et al., 2013 [9]	P300 & SSVEP	Hybrid: oddball & SSVEP	ROC thresholding using SVM scores (P300) and relative powers (SSVEP)	8 CS
Pinegger et al., 2015 [5]	P300	RCP	Thresholding using LDA scores and sum of spectral components	10 CS
Breitwieser et al., 2016 [13]	P300 and SSSEP	Hybrid: tactile & oddball	Thresholding using multi-class LDA	14 CS
Martínez-Cagigal et al., 2017 [6]	P300	RCP	ROC thresholding using LDA scores	5 CS, 16 MS
He [10]	P300	RCP	Combination of two different SVM	8 CS
Yu et al., 2017 [18,22]	P300 and MI	MI monitoring & RCP	MI signal activates the RCP	11 CS, 8 CS
Alcaide-Aguirre et al., 2017 [12,14]	P300	RCP	Certainty algorithm: t-test over LDA scores	11 CS, 19 CP
Ma & Qiu, 2018 [21]	P300	RCP	ROC thresholding using relative powers	4 CS
Aydin et al., 2018 [20]	P300	Hex-o-Spell	ROC thresholding using classifier labels	10 CS
Tang et al., 2018 [15]	P300	RCP	ROC thresholding using LDA scores	4 CS
Martínez-Cagigal et al., 2019 [16]	P300	RCP	ROC thresholding using LDA scores	18 CS, 10 MD
**Present study**	**P300**	**RCP**	**LDA classification using** **SampEn features**	**10 CS**

SSVEP: steady-state visual evoked potentials, SSSEP: somatosensory evoked potentials, MI: motor imagery, RCP: row-col paradigm, ROC: receiver operating characteristic, SVM: support vector machines, PSD: power spectral density, LDA: linear discriminant analysis, SampEn: sample entropy, CS: control subjects, MS: multiple sclerosis, CP: cerebral palsy, MD: motor-disabled.
