# Correction: Martínez-Cagigal, V.; Santamaría-Vázquez, E.; Hornero, R. Asynchronous Control of P300-Based Brain–Computer Interfaces Using Sample Entropy. *Entropy* 2019, *21*, 230

**DOI:** 10.3390/e22050505

**Published:** 2020-04-28

**Authors:** Víctor Martínez-Cagigal, Eduardo Santamaría-Vázquez, Roberto Hornero

**Affiliations:** Biomedical Engineering Group, E.T.S.I. Telecomunicación, University of Valladolid, Paseo de Belén 15, 47011 Valladolid, Spain

Figure 5 of the original paper contains errors [1]. However, the issue has no direct consequence on the manuscript, since the discussion and conclusions were made according to the corrected figure, which is shown below:

## Figures and Tables

**Figure 5:**
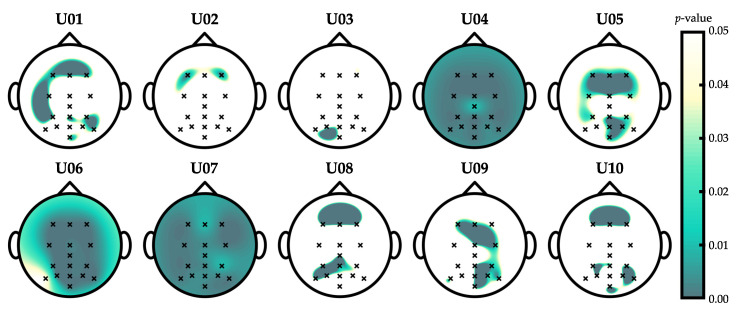
Wilcoxon signed-rank test *p*-values that show significant differences (i.e., from 0 to 0.05) between control and non-control SampEn features in the optimization dataset. Hyperparameters were fixed to their optimal values. Note that *p*-values were adjusted using the Benjamini–Hochberg False Discovery Rate (FDR) step-up procedure.
